# Identifying Differential Expression Genes and Prognostic Signature Based on Subventricular Zone Involved Glioblastoma

**DOI:** 10.3389/fgene.2022.912227

**Published:** 2022-07-08

**Authors:** Qing Yuan, Fu-Xing Zuo, Hong-Qing Cai, Hai-Peng Qian, Jing-Hai Wan

**Affiliations:** Department of Neurosurgery, National Cancer Center/National Clinical Research Center for Cancer/Cancer Hospital, Chinese Academy of Medical Sciences and Peking Union Medical College, Beijing, China

**Keywords:** glioblastoma, subventricular zone, gene expression signature, prognosis, immune infiltration

## Abstract

**Background:** Studies have suggested that glioblastoma (GBM) cells originate from the subventricular zone (SVZ) and that GBM contact with the SVZ correlated with worse prognosis and higher recurrence. However, research on differentially expressed genes (DEGs) between GBM and the SVZ is lacking.

**Methods:** We performed deep RNA sequencing on seven SVZ-involved GBMs and paired tumor-free SVZ tissues. DEGs and enrichment were assessed. We obtained GBM patient expression profiles and clinical data from the Chinese Glioma Genome Atlas (CGGA) and The Cancer Genome Atlas (TCGA) databases. The least absolute shrinkage and selection operator Cox regression model was utilized to construct a multigene signature in the CGGA cohort. GBM patient data from TCGA cohort were used for validation.

**Results:** We identified 137 (97 up- and 40 down-regulated) DEGs between GBM and healthy SVZ samples. Enrichment analysis revealed that DEGs were mainly enriched in immune-related terms, including humoral immune response regulation, T cell differentiation, and response to tumor necrosis factor, and the MAPK, cAMP, PPAR, PI3K-Akt, and NF-κb signaling pathways. An eight-gene (*BCAT1*, *HPX*, *NNMT*, *TBX5*, *RAB42*, *TNFRSF19*, *C16orf86*, and *TRPC5*) signature was constructed. GBM patients were stratified into two risk groups. High-risk patients showed significantly reduced overall survival compared with low-risk patients. Univariate and multivariate regression analyses indicated that the risk score level represented an independent prognostic factor. High risk score of GBM patients negatively correlated with 1p19q codeletion and *IDH1* mutation. Immune infiltration analysis further showed that the high risk score was negatively correlated with activated NK cell and monocyte counts, but positively correlated with macrophage and activated dendritic cell counts and higher *PD-L1* mRNA expression.

**Conclusion:** Here, a novel gene signature based on DEGs between GBM and healthy SVZ was developed for determining GBM patient prognosis. Targeting these genes may be a therapeutic strategy for GBM.

## Introduction

Gliomas are the most prevalent primary tumors of the central nervous system (Chen et al., 2017), representing approximately 80% of malignant brain tumors (Ostrom et al., 2014), and they are responsible for the majority of deaths from primary brain tumors (Weller et al., 2015). In particular, patients with high-grade glioma (WHO IV), also known as glioblastoma multiforme (GBM), have a median overall survival (OS) of 15–17 months with clinical management (Stupp et al., 2005; Gilbert et al., 2013; Bell et al., 2017). Although concurrent chemoradiotherapy followed by surgical tumor resection has become the standard treatment for GBM patients, their survival remains abysmal. Recent research has focused on the aberrant molecular alterations in gliomas. *IDH1/2* mutation (Demyashkin and Nikitin, 2018), 1p/19q codeletion (Cairncross et al., 1998), TERT promoter mutation (Arita et al., 2016) and several other markers are used to define glioma subtypes (Eckel-Passow et al., 2015). Despite improvements in therapeutic strategies, there has been no substantial improvement in glioma patient OS, especially for GBM patients, over the past decade, partially because of poorly understood mechanisms of GBM initiation and progression. Therefore, identifying cells of origin harboring novel markers that drive GBM initiation could provide a fundamental framework for understanding glioma progression and developing new treatments.

The development of the human cerebrum is a carefully orchestrated process, whereby neuronal precursor cells migrate radially from the stem cell niche in the subventricular zone (SVZ). During neural development, the SVZ is the site of extraordinary proliferation, where nearly 250,000 new neural precursor cells are generated per minute. Studies have suggested that neural stem cells (NSCs) in the SVZ of the adult and pediatric human brain, with their self-renewal and proliferative capacities, may be the cells, from which gliomas originate. This notion has been supported by direct genetic evidence that astrocyte-like NSCs in the SVZ harbor driver mutations in human gliomas, which could lead to glioma development (Alcantara Llaguno et al., 2009; Lee et al., 2018; Sanai et al., 2004; Tomasetti et al., 2017). These findings establish a clinical link between NSCs in the SVZ and the initiation and progression of glioma and suggest possible therapeutic interventions to improve patient outcomes. A pervious study showed that GBMs proximal to the SVZ exhibit mRNA expression profiles associated with stem cell properties, increased DNA repair capacity, and poor clinical survival (Steed et al., 2020). However, there is a lack of direct genetic evidence for this link in GBM patients.

Thus, the aim of the present study was to detect differentially expressed genes (DEGs) between tumor-free SVZ and SVZ-involved GBM tissues. Expression profiles and clinical data of GBM patients from the Chinese Glioma Genome Atlas (CGGA) were used as the training group to construct an RNA prognostic signature, which was validated using The Cancer Genome Atlas (TCGA)-GBM dataset. Thus, a potential new tool was developed for prognostic prediction and treatment of SVZ-involved GBMs.

## Methods and Materials

### Sample Preparation

Specimens were procured from the Department of Neurosurgery at the National Cancer Center (NCC)/Cancer Hospital of the Chinese Academy of Medical Sciences. All specimens used were residual tissues collected after diagnostic sampling. SVZ specimens were obtained from the resection margin, which was confirmed to be free of tumor cells using intraoperative frozen section evaluation. None of the patients received neoadjuvant treatment before surgery, and all signed separate informed consent forms for sample collection and molecular analysis. This study was conducted in accordance with the World Medical Association Declaration of Helsinki. Primary tumor regions from freshly excised tumor research tissues were sampled by experienced pathologists and immediately stored at –80°C. This study was approved by the Ethics Committee of the Cancer Hospital, Chinese Academy of Medical Sciences (Number NCC3410).

### Library Construction and Sequencing

Total RNA was extracted from the tissue samples using TRIzol reagent (Invitrogen, Carlsbad, CA, United States), according to the manufacturer’s instructions. Subsequently, the total RNA was quantified using a NanoDrop 2000 spectrophotometer (Thermo Scientific) and Agilent 2,100 Bioanalyzer (Agilent Technologies). Next, the RNA was reverse-transcribed using random primers to obtain cDNA, which was used for library construction. Sequencing was performed using a BGISEQ-500 system at the Beijing Genomics Institute, China. Sequencing data could be found at https://ngdc.cncb.ac.cn (NO.PRJCA009119).

### Public Database Access

Profiles of gene expression data and the relative baseline information of GBM patients were accessed from the TCGA-GBM (https://xenabrowser.net/datapages/) and CGGA (https://www.cgga.org.cn/, RNA sequencing data of mRNAseq_693 and mRNAseq_325). These two datasets were then loaded in R software (4.0.2) to correct the batch effects with the help of “limma” and “sva” packages.

### Identification of Differentially Expressed Genes and Enrichment Analysis

To eliminate the effect of genes with low expression abundance, genes with FPKM < 1 in all samples were excluded. Differential expression genes between healthy SVZ and GBM were identified using DESeq2. The significance thresholds were set as adjusted *p* value < 0.05 and |log2Foldchange| > 0.8 after Wilcoxon rank-sum test and genes meet the conditions were regarded as DEGs. A volcano and heatmap plot were created. Enrichment analysis were performed as we previously done (Yuan et al., 2021).

### Independent Prognostic Analysis Filtering

After screening for DEGs between the SVZ and GBM tissue samples, we compared this dataset with the expression of these genes in the CGGA-GBM database. Univariate Cox analysis of OS were then performed to screen the SVZ-related genes with prognostic values. A *p* value < 0.001 was treated as statistically significant. The calculation of SVZ-related genes was performed in R using “survival” and “survminer” packages.

### Construction and Validation of the Risk Predictive Model

Using Cox regression with the least absolute shrinkage and selection operator (LASSO) method, a DEG signature to predict GBM patient prognosis was constructed using the “glmnet” package in R. Lambda.1se was selected after 10,000 times ten-fold cross-validation to prevent the overfitting effects of the model. The following formula was used to evaluate the risk score of GBM patients:

$$e^{\left(each\;genes\;\exp ression\times corresponding\;coeffecient\right)}$$ (1)

According to the median risk score of the novel signature, the GBM patients were separated into high- and low-risk groups. Additionally, for the test group (TCGA-GBM), we calculate the risk score using the same formula to verify the accuracy of the signature.

### Kaplan–Meier Survival Analysis and Predicting Nomogram Construction

Survival analysis was performed to examine the accuracy of the SVZ-related signature by dividing GBM patients into differential risk groups. OS curves of GBM patients were then created using the K–M method, and the log-rank was taken in the comparisons. To examine the correlations between the risk score of our novel signature and clinic parameters including gender, age, treatment, and other molecular alterations, the χ^2^ test was used. Finally, to predict the survival of GBM individuals at 12, 24, and 36 months after surgery, a nomogram model, including the risk score of SVZ-related signature, was established using the “rms” package as we previously down (Yuan et al., 2021).

### Immune Infiltration Analysis

Relationship between the risk score of the SVZ-related signature and tumor-infiltrating immune cells (TIICs) was analyzed. The transcript expression data of GBM tissues were loaded in R and rate of 21 TIICs were calculated using the “CIBERSORT” package. After that, we compared the difference in containment for all the TIICs between different risk score groups using Spearman’s test. To examine the accuracy of the results, we re-tested the relationship between the proportion of immune cells with the risk level using the xCell method (Aran et al., 2017).

## Results

### Study Design

**FIGURE 1:**
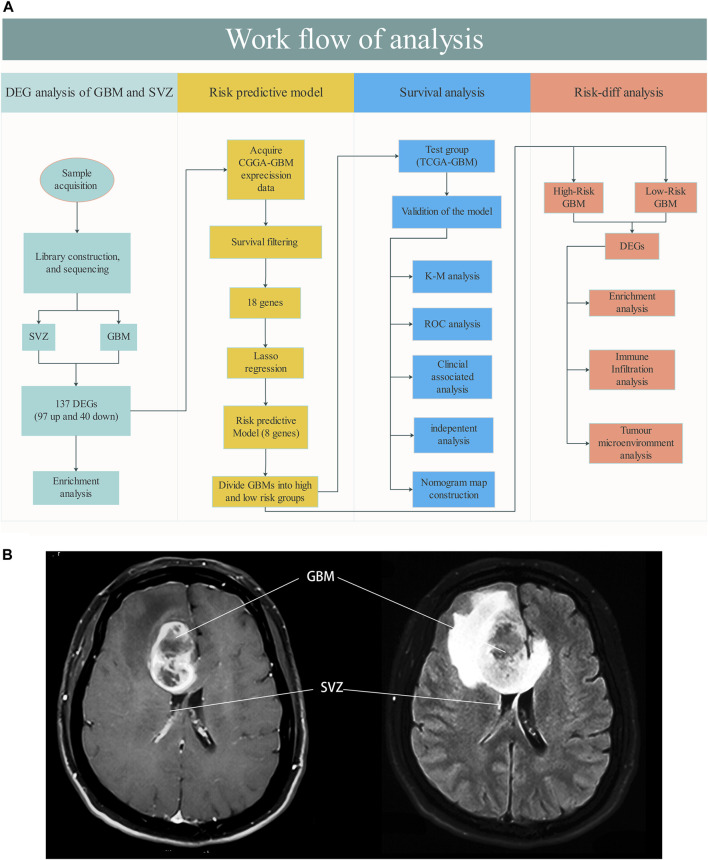
**(A)** Workflow of the analysis. **(B)** Schematic diagram about SVZ involved GBM (MRI T1 Flair).

A flow chart of the study design is shown in Figure 1A. Seven GBM and six paired SVZ tissues (one was excluded due to low quality) from the NCC were used for library construction and sequencing. After excluding cases with incomplete survival and clinical information, the data of the remaining GBM patients from the public databases (290 CGGA patients and 226 TCGA patients) were included in subsequent analyses. The baseline clinical information of the patients is shown in Table1.

**TABLE1:** Baseline information of patients with glioma in CGGA and TCGA.

Variables	CGGA				TCGA			
	Total	Risk^High^	Risk^Low^	*p* value	Total	Risk^High^	Risk^Low^	*p* value
Gender								
Male	176	80 (45.5%)	96 (54.5%)	0.071	126	73 (57.9%)	53 (42.1%)	0.007*
Female	114	65 (57.0%)	49 (43.0%)		100	40 (40.0%)	60 (60.0%)	
Age (years)								
≤50	86	41 (47.7%)	45 (52.3%)	0.607	78	26 (33.3%)	52 (66.7%)	<0.001*
>50	204	104 (51.0%)	100 (49.0%)		148	87 (58.8%)	61 (41.2%)	
Radiotherapy								
Yes	247	122 (49.4%)	125 (50.6%)	0.741	152	82 (53.9%)	70 (46.1%)	0.288
No	43	23 (53.5%)	20 (46.5%)		61	28 (45.9%)	33 (54.1%)	
NA					13	3 (23.1%)	10 (76.9%)	
Chemotherapy								
Yes	244	125 (51.2%)	119 (48.8%)	0.335	150	78 (52.0%)	72 (48.0%)	0.489
No	46	20 (43.5%)	26 (56.5%)		62	29 (46.8%)	33 (53.2%)	
NA					14	6 (42.9%)	8 (57.1%)	
Pathology								
Primary	175	93 (53.1%)	82 (46.9%)	0.242		NA	NA	
Secondary	90	41 (45.6%)	49 (54.4%)					
Recurrent	25	11 (44.0%)	14 (56.0%)					
IDH mutation								
Mutant	67	16 (23.9%)	51 (76.1%)	<0.001*	59	3 (5.1%)	56 (94.9%)	<0.001*
Wildtype	223	129 (57.8%)	94 (42.2%)		159	104 (65.4%)	55 (34.6%)	
NA					8	6 (75%)	2 (25%)	
1p19q_codeletion								
codeletion	15	2 (13.3%)	13 (86.7%)	<0.001*		NA	NA	
No-codeletion	275	143 (52.0%)	132 (48.0%)					
living status								
live	42	17 (40.5%)	25 (59.5%)	<0.001*	73	23 (31.5%)	50 (68.5%)	<0.001*
dead	248	128 (51.6%)	120 (48.4%)		153	90 (58.8%)	63 (41.2%)	
Total	290	145(50%)	145(50%)		226	113(50%)	113(50%)	

*Statistically significant difference (*p* value < 0.05) and same below.

NA, not available; CGGA, chinese glioma genome atlas; TCGA, the cancer genome atlas; WHO, world health organization; GBM, glioblastoma.

### Differentially Expressed Genes and Enrichment Analysis Between Healthy Subventricular Zone and Glioblastoma Tissues

**FIGURE 2:**
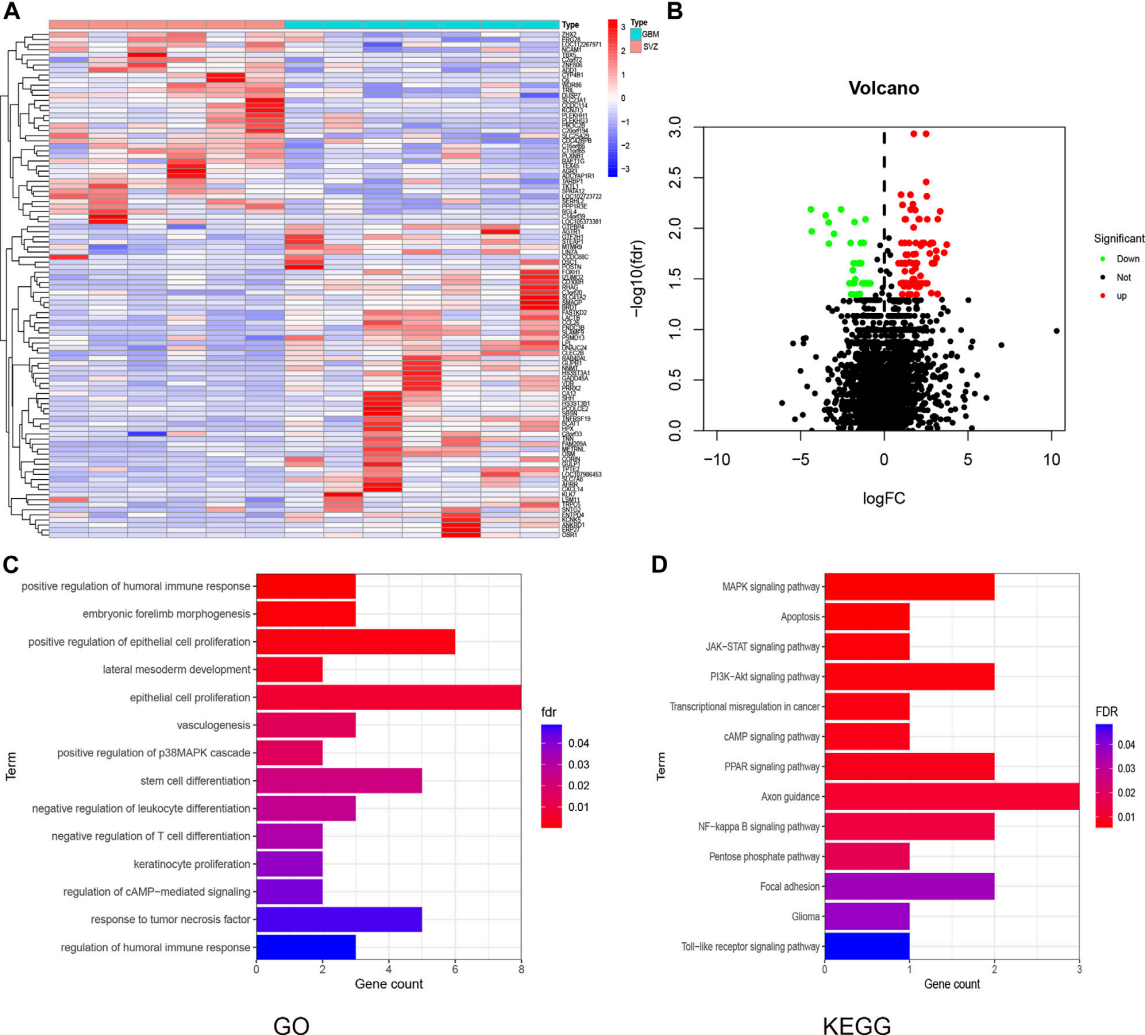
DEG and enrichment analysis for SVZ and GBM. **(A)** Heatmap for DEGs generated by comparison of the SVZ vs. GBM. Row name of headman is the gene name, and the column name is the ID of samples which not showed in a plot. **(B)** Volcano map of distribution of DEGs. Red point shows up-regulated DEGs while green shows down-regulated in GBM compared with SVZ. **(C,D)** GO and KEGG analysis for these DEGs in GBM.

We compared the gene expression differences in the DEGs between healthy SVZ and GBMs using the DESeq2 package. A total of 97 and 40 upregulated and downregulated DEGs were identified, respectively. The expression patterns of the 20 genes with the largest increase or decrease between groups were visualized as a heatmap (Figure 2A). DEG distribution was visualized using a volcano plot (Figure 2B).

Gene Ontology (GO) enrichment analysis revealed that DEGs were enriched in various immune-related terms, including humoral immune response regulation, T cell differentiation, and response to tumor necrosis factor (Figure 2C). Kyoto Encyclopedia of Genes and Genomes (KEGG) pathway analysis showed that the DEGs were mainly enriched in the MAPK, cAMP, PPAR, PI3K-Akt, and NF-κb signaling pathways (Figure 2D).

### Construction and Verification of the Risk Prognostic Signature

**FIGURE 3:**
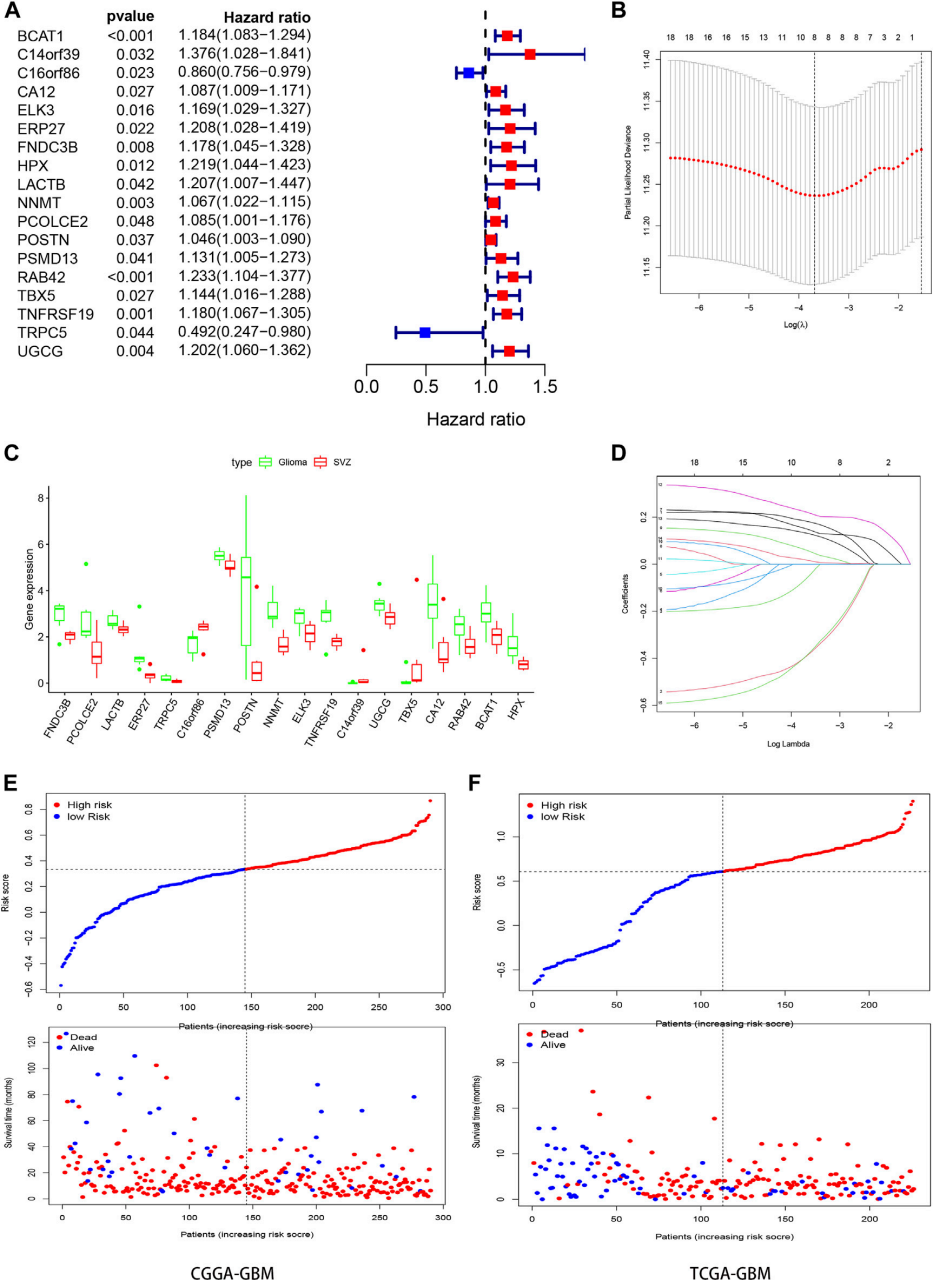
Construction the SVZ-related prognostic signature. **(A)** Forest plots showing the results of the univariate Cox regression analysis between gene expression and OS. **(C)** Boxplot showed the expression of these genes in SVZ (red) and GBM (green). **(B,D)** Lasso regression identifies 8 genes (BCAT1, C16orf86, HPX, NNMT, RAB42, TBX5, TNFRSF19, and TRPC5) in the prognostic signature for GBM and the coefficients of the signature. **(E,F)** Classification of GBM patients in CGGA and TCGA into high- and low- risk groups based on the median of risk score.

We evaluated the expression of 137 DEGs in the CGGA-GBM database. After survival and Cox regression filtering, 18 genes were selected (Figure 3A). The differential expression of these genes between healthy SVZ and GBM tissues was plotted in a boxplot (Figure 3C). These genes were used to establish a risk score using the LASSO logistic regression model (Figures 3B,D). Finally, eight genes were selected to construct the signature. Among them, six (*BCAT1*, *HPX*, *NNMT*, *TBX5*, *RAB42*, and *TNFRSF19*) were prognostic risk factors and two (*C16orf86* and *TRPC5*) were prognostic protective factors. The formula used was as follows:

Risk score = [(*BCAT1*)^∗^ (0.128907)] + [(*C16orf86*)^∗^ (−0.319,874)] + [(*HPX*)^∗^ (0.160926)] + [(*NNMT*)^∗^ (0.033013)] + [(*RAB42*)^∗^ (0.202202)] + [(*TBX5*)^∗^ (0.110279)] + [(*TNFRSF19*)^∗^ (0.006332)] + [(*TRPC5*)^∗^ (−0.307717)]. (2).

The patients from the training and test groups were divided into high- and low-risk groups separately according to the median risk score values calculated using this formula, and the survival status of GBM patients in each group is visualized in Figures 3E,F.

**FIGURE 4:**
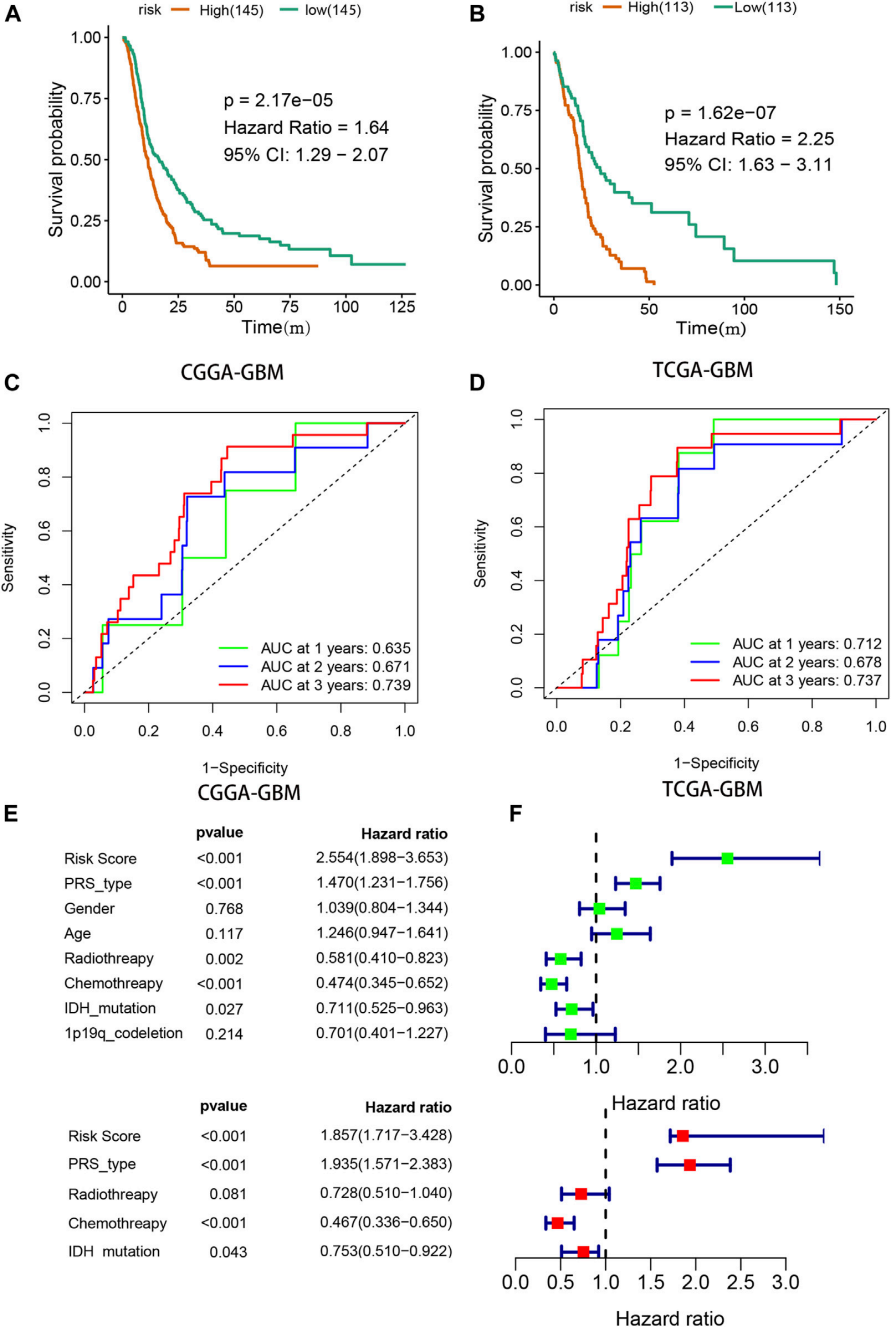
Validation of prognostic signature. **(A,B)** Survival analysis for high- and low- risk GBM patients in CGGA and TCGA. **(C,D)** Receiver operator characteristic curve analysis of signature. AUC, area under the curve. (**E,F**) Univariate and multivariate analysis of risk signature.

Kaplan-Meier analysis with the log-rank test indicated that the OS of GBM patients in the high-risk group was lower than that of patients in the low-risk group in both the CGGA and TCGA groups [(9.5 vs. 13.7 mo), HR (95% CI) = 1.64 (1.29–2.07) in the CGGA cohort, and 7.5 vs. 13.5 mo, HR (95% CI) = 2.25 (1.63–3.11) in TCGA cohort, respectively, both *p* < 0.001, Figure 4A,B]. ROC curve analysis was used to evaluate the prognostic accuracy of the model, and the areas under the curve (AUCs) in 1, 3, and 5 years were 0.635, 0.671, and 0.739, respectively, in the CGGA-GBM group (Figure 4C). The AUCs for the TCGA-GBM group were 0.712, 0.678, and 0.734, respectively (Figure 4D). Univariate Cox analyses suggested that radiotherapy, chemotherapy, PRS type (including primary, secondary, and recurrency), IDH mutation status, and the risk score of our signature correlated with GBM patient survival (Figure 4E). Multivariate Cox analyses indicated that PRS type, chemotherapy, and risk score were independent survival indicators for GBM patients (Figure 4F).

### Relationship Between Subventricular Zone-Glioblastom Signature and Clinicopathological Features

**FIGURE 5:**
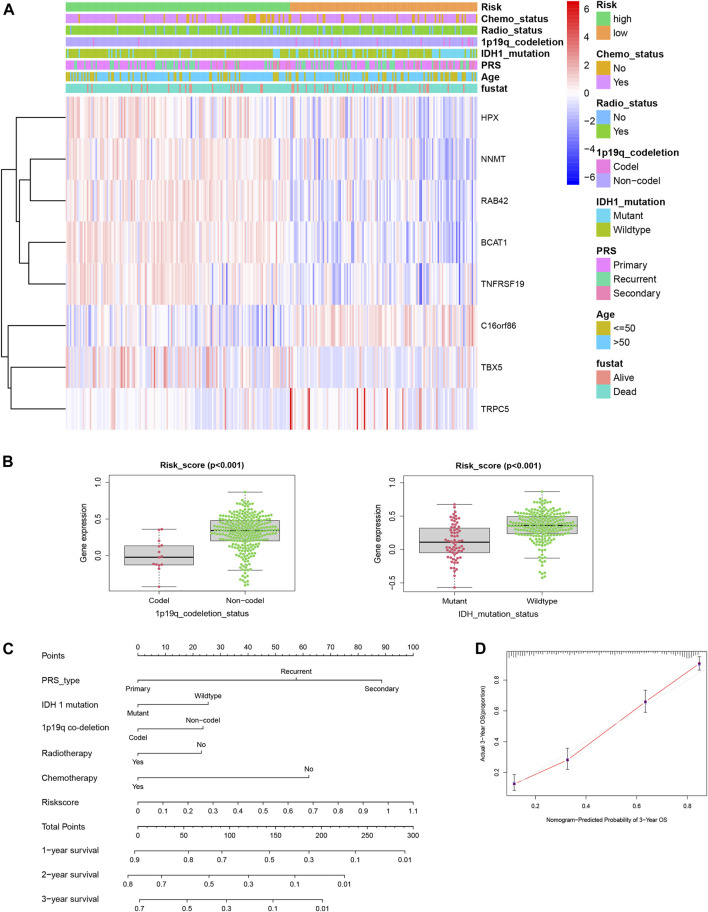
**(A)** Distribution of the risk score, the clinical parameters and the signature mRNA expression heat map in the TCGA dataset. **(B)** Risk score of GBM patients was significantly related to 1p19q co-deletion status (Codel, *n* = 15; Non-codel, *n* = 275, *p* < 0.001) and IDH1 mutation status (mutation, *n* = 67; wildtype, *n* = 223, *p* < 0.001). **(C)** Nomogram showed the prediction of survival possibilities for GBM patients in 1, 2, and3 years. **(D)** Nomogram-predicted probability of 3-year OS in GBM patients.

To obtain more complete clinical information, we used the CGGA-GBM database to explore the relationship between the genes in the predictive signature and the clinical characteristics. The expression patterns of the eight genes in the different clinicopathological GBM patient groups were plotted on a heatmap (Figure 5A). The correlation between the final risk score of each patient and their clinical features was examined, and high risk scores were strongly negatively correlated with codeletion of 1p19q chromosomes and *IDH1* mutation (both *p* < 0.001, Figure 5B). As shown in Figure 5C, we used the clinical characteristics, which served as independent survival indicators, and risk scores to construct a new nomogram with the “rms” package in the R software to predict the survival time of glioma patients more precisely and to validate the results (Figure 5D).

### Biological Function Analysis of GBM Based on Risk Score

**FIGURE 6:**
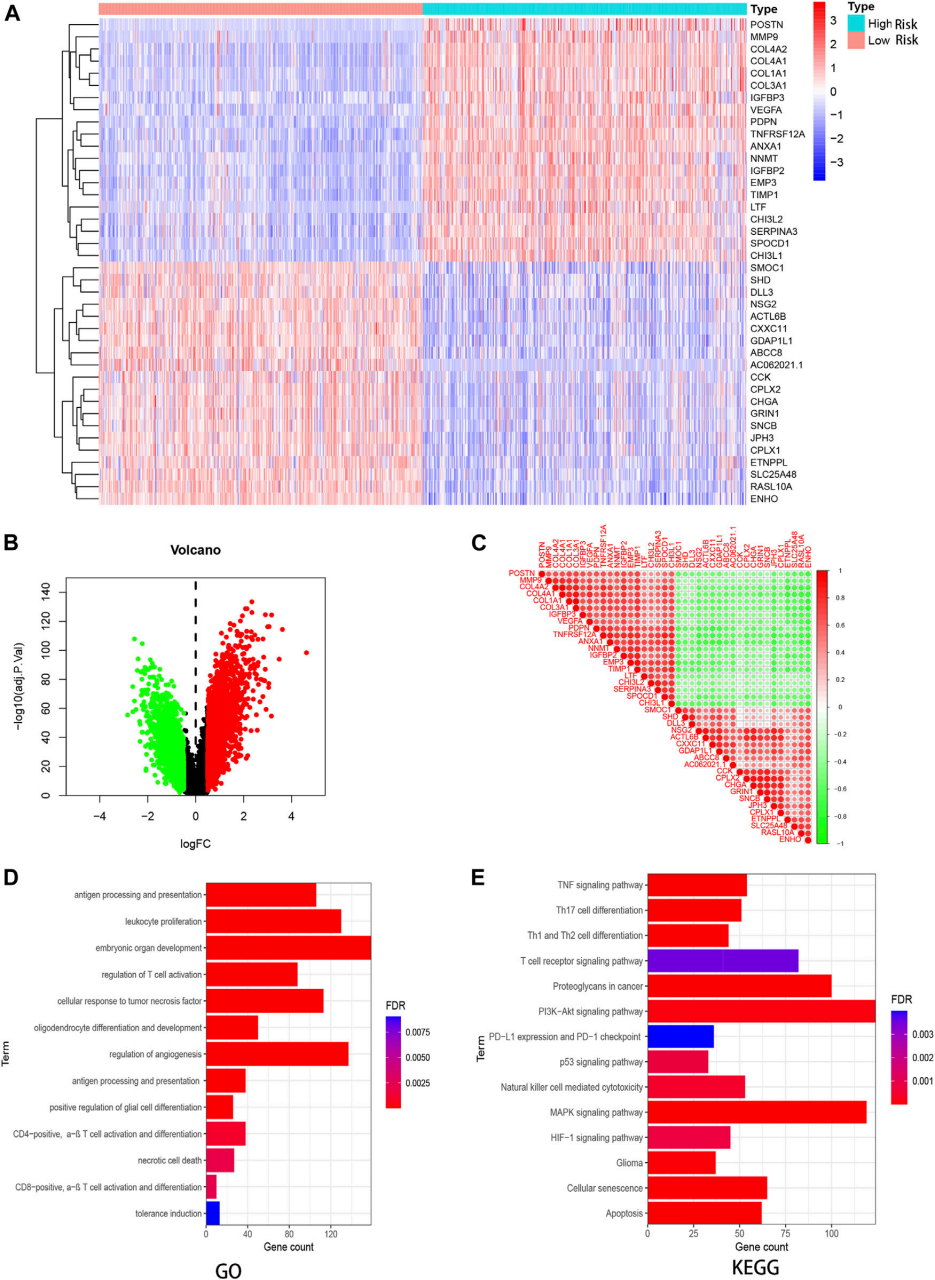
DEG and enrichment analysis for risk signature. **(A)** Heatmap for DEGs generated by comparison of the high- and low- risk individuals. **(B)** Volcano map of distribution of DEGs. **(C)** Heatmap of correlations between differential genes. Red circles represent positively correlation, green represents negative. **(D,E)** GO and KEGG analysis for these DEGs in GBM.

By dividing GBM patients into high- and low-risk score groups, we compared the gene level differences between them and found 3,475 upregulated and 1825 downregulated genes (Figure 6B). The expression patterns of the 20 upregulated and 20 downregulated genes with the largest differences in GBM individuals were plotted on a heatmap (Figure 6A). The correlation between any two of these 40 genes is shown in Figure 6C.

Based on the DEGs, we performed GO and KEGG analysis. The DEGs were mainly enriched in immune and invasion-related terms, including antigen processing and presentation, regulation of T cell activation, necrotic cell death, and a series of signaling pathways, including MAPK, TNF, PI3K-Akt, and p53.

### Analysis of Immune Infiltration Level and the Tumor Microenvironment

**FIGURE 7:**
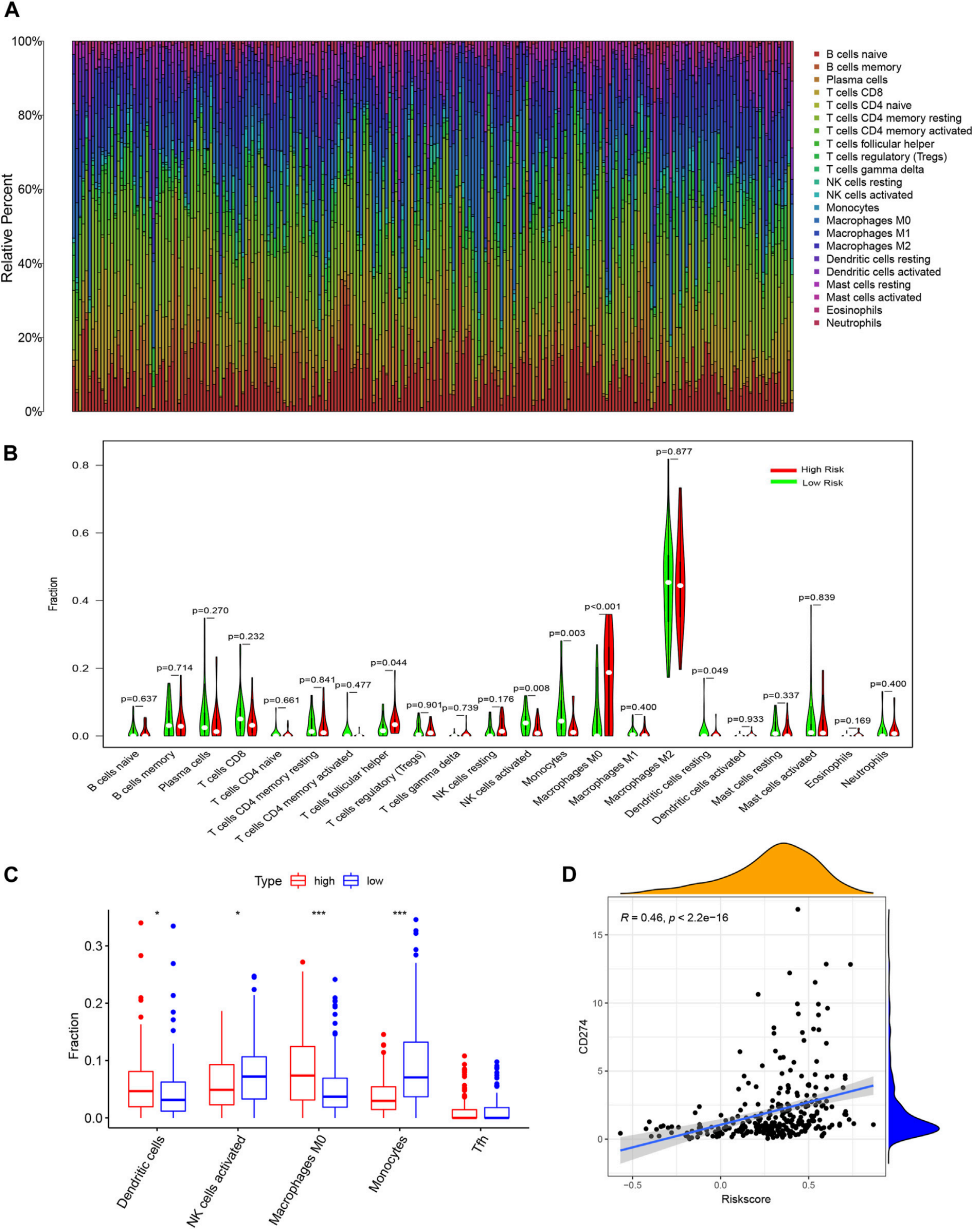
Analysis of risk-signature in immune-related activities of glioma. **(A)** Barplot showing the proportion of 21 kinds of tumor-infiltrating immune cells (TICs) in CGGA GBM samples (calculating through CIBERSORT). **(B)** Violin plot showed the ratio differentiation of 21 kinds of immune cells between CGGA glioma samples with low or high risk score. **(C)** Fraction of immune cells examined through xCell methods. **(D)** Correlation between level of CD274 (PD-L1) and riskscore.

We further analyzed the relationship between risk scores and TIICs. The comparative content of immune cell types in each case was represented using a bar plot (Figure 7A). We investigated the differences in immune cell proportions between the high-risk and low-risk subgroups. High risk score was negatively correlated with activated NK cell and monocyte counts (*p* = 0.008 and 0.003, respectively), but positively correlated with T follicular helper (Tfh) cell, M0 macrophage, and activated dendritic cell counts (*p* = 0.044, <0.001, and 0.049, respectively, Figure 7B). The xCell results showed a similar conclusion, and only the Tfh fraction was no longer correlated with the risk score level (Figure 7C). Furthermore, to explore the potential of the signature in immune therapy, we examined the relationship between risk score with *PD-L1 (CD274)* transcription level, and the result showed that *PD-L1* expression was positively correlated with risk score (R = 0.46, *p* < 0.001).

## Discussion

GBM is the most malignant form of primary brain tumor found in clinical practice, associated with extremely poor prognosis. The high malignancy and frequent recurrence of SVZ-involved GBM suggest an underlying mechanism of GBM development. Here, we explored DEGs at the transcriptome level. First, we performed deep RNA sequencing on seven SVZ-involved GBMs and paired tumor-free SVZ tissues to identify DEGs. We obtained the expression profiles and clinical data of GBM patients from the CGGA database for these DEGs. LASSO and Cox regression models were used to construct an eight-gene signature to predict the survival of GBM patients, which was validated in TCGA-GBM database. Further analysis showed that high risk score negatively correlated with 1p19q codeletion and *IDH1* mutation, which was linked to the counts of multiple immune cells in the GBM microenvironment. Thus, our findings may provide a potential new tool for prognostic prediction and treatment of SVZ-involved GBMs.

Recent studies have established the critical role of the SVZ in glioma development. Human SVZ has been found to contain NSCs (Sanai et al., 2004). Using both genetic and stereotactic injection approaches, researchers demonstrated that adult NSCs can give rise to malignant astrocytoma *in vivo* (Alcantara Llaguno et al., 2009). In addition, Lee et al. discovered that astrocyte-like NSCs with driver mutations spread from the SVZ and lead to the development of GBM in remote regions of the brain using single-cell sequencing and laser microdissection analyses of patient brain tissue and genetic modification in a mouse model (Lee et al., 2018). These findings strongly suggest that GBM originates from the SVZ. A recent clinical trial demonstrated that direct contact of GBM with the SVZ could serve as an independent predictor of shorter survival and correlated with more aggressive recurrence (Comas et al., 2021). Therefore, the identification of specific markers and their roles in SVZ-involved GBM is essential to patient management. Since the SVZ is located deep inside the brain, it is hard to reach through traditional surgical routes. In addition, inappropriate ventricle opening might cause tumor cells to spread via the ventricular circulation system. Thus, SVZ tissue is difficult to acquire. Here, we used residual tissues collected after diagnostic sampling, which was confirmed by pathologists as free of tumor cells, as the control for GBM tissues, and the analyses were performed subsequently.

In the present study, we systematically explored gene expression at the transcript level and identified the biological processes associated with SVZ-involved GBM. The DEGs between healthy SVZ and GBM were mainly enriched in tumor-related pathways, such as the PI3K-Akt, NF-κb, and JAK-STAT signaling pathways, which are strongly correlated with the occurrence and development of glioma and immune-related functions, including regulation of T cell differentiation and humoral immune response (Aldape et al., 2015; Mao et al., 2012). Remarkably, we found that these DEGs were also enriched in oligodendrocyte differentiation and development, suggesting that these genes contributed to the progression of glioma cell development and invasion.

Through survival and Cox regression analysis, we selected 18 genes for the construction of the risk model. Among them, *PCOLCE2*, *ERP27*, *PSMD13*, *C14orf39*, *C16orf86*, *UGCG*, and *HPX* were identified as prognostic factors for glioma for the first time. *PCOLCE2* has been identified as a biomarker in multiple cancers, including colorectal and gastric cancers (L. Chen L et al., 2019; Xu et al., 2021). *ERP27* is highly expressed in colorectal cancers, and its expression is correlated with patient survival (Yu et al., 2021). *PSMD13* expression in lung adenocarcinoma strongly correlates with *PSMC6* expression, which is associated with poor tumor differentiation and might act as a therapeutic target in lung adenocarcinoma (Zhang et al., 2021). *UGCG* influences glutamine metabolism in breast cancer cells (Schömel et al., 2019). The HPX molecule is capable of binding heme with high affinity, acting as a heme-specific carrier from the bloodstream to the liver (Tolosano et al., 2010). Experimental and epidemiological studies have shown that free oxygen radicals play an important role in cancer pathogenesis, including gliomas (Chen Y et al., 2019). We hypothesized that HPX was overexpressed due to oxidative stress buildup during the progression of glioma development, as it enacts a preventive role against oxidative damage through cellular defense mechanisms. Further mechanistic experiments and studies involving a larger number of patients should be performed to validate the function and prognostic role of these markers.

Finally, our findings enabled the construction of a gene expression signature comprising eight genes—*BCAT1*, *C16orf86*, *HPX*, *NNMT*, *RAB42*, *TBX5*, *TNFRSF19*, and *TRPC5*—that were strongly associated with the clinical characteristics of GBM and showed good prognostic accuracy in both the CGGA and TCGA datasets. *IDH1* mutation and 1p19q codeletion status have been widely used in pathological diagnosis and survival prediction in GBM patients. Previous studies showed that BCAT1 could inhibit glutaminase-specific sensitization of *IDH*-mutant glioma cells to oxidative stress *in vitro* and to radiation *in vitro* and *in vivo* (Tönjes et al., 2013; Chaumeil et al., 2014; McBrayer et al., 2018). Thus, overexpression of BCAT1 was associated with poor prognosis in GBM patients (Cho et al., 2017; Chen Y Y et al., 2019). In agreement with this finding, in the present study, the expression of *BCAT1* mRNA was strongly correlated with *IDH1* mutation status, serving as a risk factor in survival prediction. Furthermore, a nomogram based on PRS type, chemotherapy, and the risk score of our signature were able to accurately predict the individual survival of patients with GBM.

Accumulating evidence has demonstrated the importance of the tumor microenvironment in tumor development, and TIICs can serve as promising indicators of therapeutic effects (Gajewski et al., 2013). A reduced number of inflammatory cells in the glioma microenvironment may prevent efficient immune surveillance (Price et al., 2021). Tumor-associated macrophages recruited into the glioma tumor microenvironment can release growth factors and cytokines in response to cancer cell activity and thus promote an immunosuppressive phenotype in glioma cells (Domingues et al., 2016; Hambardzumyan et al. 2016). Dendritic cells are antigen-presenting cells that are responsible for the recognition of pathogens at the site of inflammation, favoring the development of regulatory T cells in glioma (Kim et al., 2018). NK cells can modulate their antitumor function through a balance of activating and inhibitory ligands on their cell surface. Cancer immunotherapies using NK cells show immense potential for the treatment of several solid tumors, including GBM (Lupo and Matosevic, 2020). We found that the fraction of these immune cells in GBM correlated with the risk score of our signature, which suggested its potential application for targeted therapy in GBM. In addition, anti-PD-L1 antibodies have demonstrated powerful antitumor effects in multiple cancers. However, owing to the specific cellular and structural microenvironment in the brain and the relationship between PD-L1 and T cell infiltration in glioma, the therapeutic effects of anti-PD-1/PD-L1 antibodies remain unclear (Huang et al., 2017). Here, we demonstrated that genes in our signature were enriched in terms of PD-L1 expression and PD-1 checkpoint pathways in cancer. Additionally, the risk score of GBM patients strongly correlated with PD-L1 transcript level, suggesting that these genes may be involved in the PD-L1-mediated immune escape pathway.

## Conclusion

In the present study, we analyzed DEGs between healthy SVZ and SVZ-involved GBM and proposed a novel eight-gene panel and nomogram to predict the survival of patients with GBM, which may contribute to more accurate prognostic prediction and better clinical decision-making for GBM patients. Further analysis revealed that these eight genes may be related to the development of glioma cells and their involvement in immune suppression in the tumor microenvironment. This study provides a theoretical basis for the future design of immune therapies based on these eight genes. A larger volume of samples and mechanistic studies were needed to validated the function of these genes in GBM.

## Data Availability

The original contributions presented in the study are publicly available. This data can be found here: https://ngdc.cncb.ac.cn; accession number PRJCA009119.
