# Supplementary material for: Identifying Differential Expression Genes and Prognostic Signature Based on Subventricular Zone Involved Glioblastoma
Source: Front Genet. 2022 Jul 8;13:912227. doi: 10.3389/fgene.2022.912227 (PMC9305325; doi:10.3389/fgene.2022.912227)

1. Quality control details of RNA-seq analysis


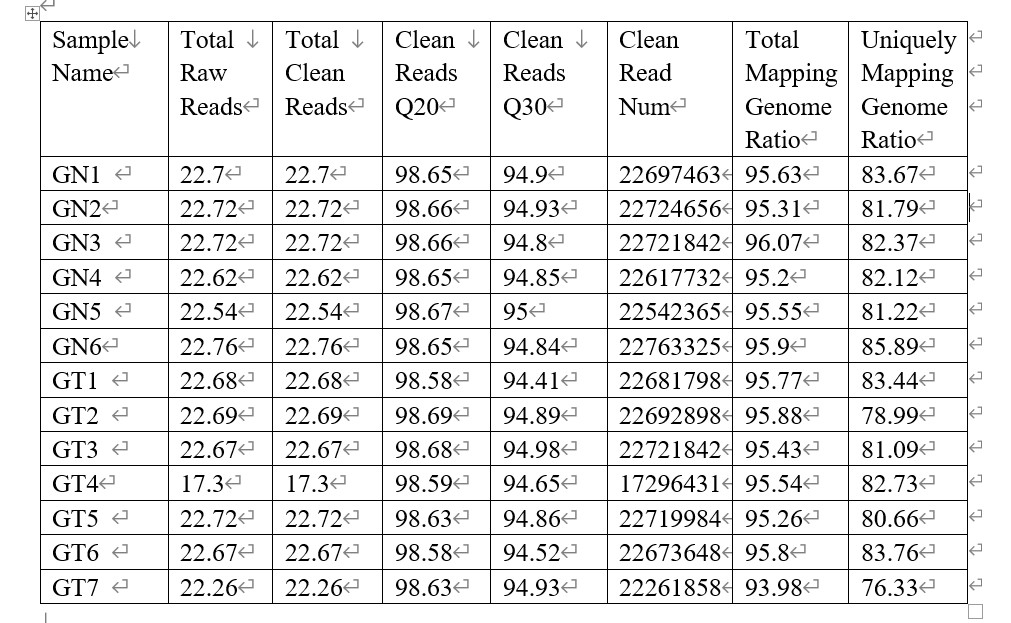


2. GO and KEGG analysis only with up regulated genes only.


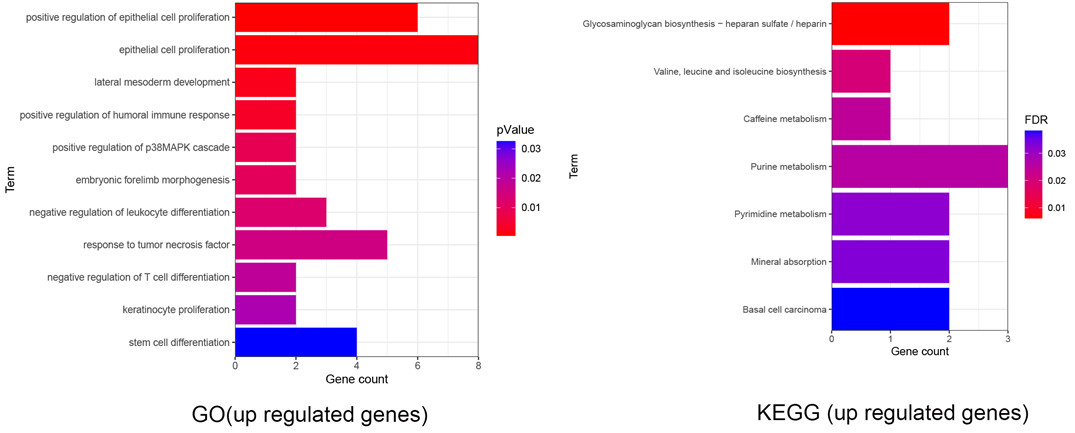

Supplement: Supplementary file 1 [file Table2.DOCX]
